# High burden of pulmonary tuberculosis and missed opportunity to initiate treatment among children in Kampala, Uganda

**DOI:** 10.4314/ahs.v22i4.66

**Published:** 2022-12

**Authors:** Samuel Kizito, Rita Nakalega, Dorothy Nampijja, Collins Atuheire, Geofrey Amanya, Edrisa Kibuuka, Hellen Nansumba, Ekwaro Obuku, Joan Kalyango, Charles Karamagi

**Affiliations:** 1 Clinical Epidemiology Unit, School of Medicine, College of Health Science Makerere University, P.O.Box 7072 Kampala; 2 Makerere University-Johns Hopkins University (MU- JHU) Research Collaboration Kampala, Uganda; 3 Mulago National Referral and Teach Hospital, P. O. Box 7051, Kampala

**Keywords:** pediatric, pulmonary, tuberculosis

## Abstract

**Background:**

There is uncertainty about the actual burden of childhood TB in Uganda, but underestimation is acknowledged. We aimed at determining prevalence, factors associated with PTB among children attending PHC facilities in Kampala.

**Methods:**

This was a cross-sectional study of 255 children, with presumed TB, attending six health facilities in Kampala, Uganda, in March 2015. Socio-demographic, clinical, and laboratory data were collected using a questionnaire. TB was diagnosed using “Desk Guide” algorithms. Sputum based on ZN/FM and/or Gene-Xpert. Logistic regression was used to assess associations with outcomes.

**Results:**

Overall, prevalence of PTB 13.7 % (2.6 – 24.8). Among HIV-positive, the prevalence of PTB was 41.7%, while among malnourished children, 21.7% and contacts, 89.3%. The factors that influenced PTB included: tobacco smoker at home (OR = 1.6, 95 % CI: 1.07 – 6.86), stunting (OR = 2.2, 95 % CI: 1.01 – 4.15). Only 5.3% of the smear-negative TB children and 81.3% of the smear-positive children were initiated on treatment within a month of diagnosis.

**Conclusion:**

Clinical TB among children is underdiagnosed and undertreated. There is a need for more sensitive and specific diagnostic tests, need ways to disseminate and promote uptake of standardized clinical algorithms. Also, contact TB tracing should be strengthened so that such cases can be actively detected even at community level.

## Introduction

Although curable, tuberculosis (TB) continues to cause high morbidity and mortality among children below 15 years 1–3. In 2019 alone, there was an estimated 1.2 million children suffering from TB, and a total of over 23,000 children died from TB 4. Despite this high burden, childhood TB has been neglected^5^–^8^ in Uganda, one of the 30 countries identified by WHO as having the highest burdens of TB 4, 9, 10.

Although the majority of childhood pulmonary tuberculosis (PTB) is smear-negative 11, 12, in Uganda, its diagnosis is mainly based on sputum AFB smear microscopy or GeneXpert MTB/RIF with limited clinical diagnosis 13, 14. Children under 15 represent less than 10% of AFB smear-positive patients notified to the national TB program 10, 14. Clinical algorithms for diagnosing of pediatric TB are not widespread and their impact has not been assessed 15. Therefore, the burden of disease in children is underestimated 16, 17. Our study aimed to determine the burden and predictors of TB, and how a standardized approach to clinical diagnosis (using the Union Pediatric TB Desk Guide) might impact case notification and treatment rates among older children in Uganda.

## Methods

### Study sites and setting

A cross-sectional study was conducted in six primary health facilities within Kampala district. Kampala is the capital city and is in central Uganda. The city has several slum areas served by these clinics. The health directorate of Kampala Capital City Authority (KCCA) provides health services to the population through 8 health facilities. These facilities receive patients directly from the community and offer outpatient and maternity inpatient services. Diagnosis of pulmonary TB is based on sputum analysis using ZN, FM, or GeneXpert MTB/RIF (Xpert) and follows the guidelines laid out in the National TB and Leprosy Program 18. Occasionally, a clinical diagnosis of PTB is made based on clinical and radiological findings for the participants who had negative sputum or were unable to expectorate 18. Routinely, the nurses usually treat TB infected children at the TB clinics, and only complicated cases are referred to clinicians.

### Sample size and participants

Based on childhood TB prevalence of 10.7% from the national TB notification rates for 2014, a target power of 80% and a precision of 0.05, we estimated a sample size of 147 children with presumed TB. We applied a design effect of 2 to yield 294 participants to account for clustering within health centers. The study consecutively enrolled children 5 to 15 years who attended outpatient departments at the KCCA clinics between February and April 2015 and had clinical features suggesting pulmonary TB, as elaborated in the intensified case finding guidelines 19. The total number of children who attended the outpatient department for the study period was obtained by reviewing the facility records. We excluded very sick children who warranted immediate referral and those below 5 years of age because clinicians routinely refer them out of the facility for further evaluation because they are usually unable to expectorate. Participants were screened from the OPD using the Intensified TB case finding form. All the eligible presumptive TB patients were then enrolled in the study.

### Study exposure variables and measurements

After pre-testing, an interviewer administered a semi-structured questionnaire to capture age, sex, nationality, religion, tribe, participant and caretaker education level, and nationality. House-hold characteristics including monthly income, persons at home, number of rooms and ventilation, energy source for cooking and cooking area. Clinical history including BCG vaccination, contact with a confirmed TB patient, IPT, ART status, chronic cough, haemoptysis, weight loss, frequent evening fever and drenching night sweats.

### HIV diagnosis

Clinics performed routine HIV counselling and testing using whole blood assays according to a parallel rapid testing algorithm recommended by the Uganda Ministry of Health 20.

### Anthropometric measurements

Weight was determined using calibrated SECCA scales; and height was measured using a non-stretching meter that was placed and calibrated against a vertical wall. Stunting, underweight, and low BMI were defined as Z-score <-2SD for height-for-age, weight-for-age, and BMI-forage respectively. These were calculated using ENA® (Emergency Nutritional Assessment) and AnthroPlus® developed by WHO. The children's nutritional status was categorized into good or bad basing on whether they had stunting and wasting or not.

### Outcome variables

Microbiological diagnosis of TB followed Uganda NTLP guidelines 21, which follow WHO recommendations on sputum smear microscopy and Xpert 22. It was based on having the spot, morning or both sputum smears positive for by microscopy or Xpert. Sputum examination analysis was done at the KCCA health facility laboratories, however two of the facilities lacked Xpert facilities. These laboratories participate in an external quality assessment program overseen by the Uganda National TB Reference Laboratory. A clinical TB diagnosis was retrospectively made from the data collected basing on the *“Desk-Guide for Diagnosis and Management of Tuberculosis in Children”* guidelines 18: having history of contact and clinical features suggestive of PTB. After a month, TB registers were reviewed in each of the facilities to determine the number of the children diagnosed in the study that were initiated on anti-TB medicine.

### Data management and analysis

Data was double-entered Epidata, validated and exported to STATA version 12.0 for analysis, adjusted for clustering at the level of the health facility. Continuous variables were summarized using measures of central tendency. Categorical variables were summarized using frequencies, proportions, and percentages. Bivariate logistic regression analysis was performed and all variables with a P-value of ≤ 0.2 were entered in a multivariate logistic regression model to assess their association with the outcomes. Interaction between the variables was assessed using the Chunk test. Confounding was assessed using a difference of ≥ 10% between the crude and adjusted odds ratio for the variables.

### Ethical Considerations

Institutional approval for this study was obtained from the Makerere University School of Medicine Research and Ethics Committee. Written informed consent was obtained from the mothers/ caregivers. Additional assent was sought from children 8 years and above. Permission was sought from the KCCA directorate of public health to conduct the study within the KCCA health facilities.

## Results

**Figure 1:**
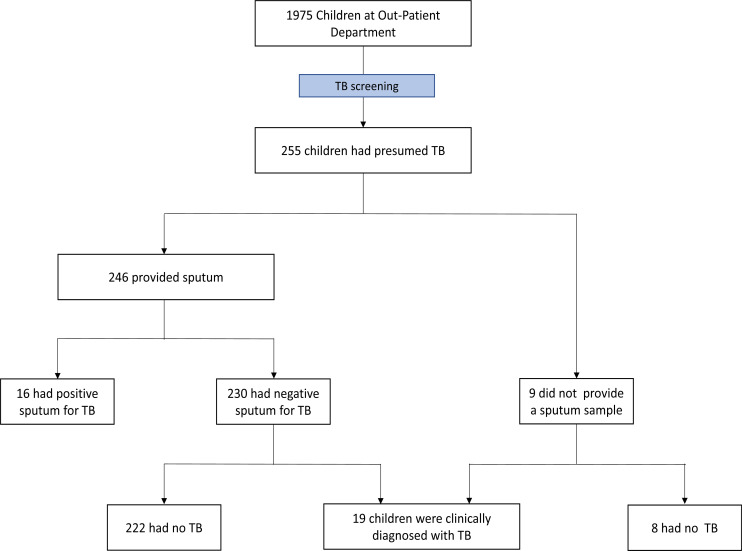
patient flow chart for children aged 5 to 14 years in six peripheral facilities in Kampala Uganda, 2015.

There were a total of 1975 children aged 5 to 14 years who attended the outpatient department during the study period. Of these, 255 had features suggestive of Tuberculosis. The patient flowchart shows details in figure 1.

### Socio-demographic characteristics

We enrolled 255 children into the study. Majority (233, 91.4%) of the participants were Ugandans, and most (238, 93.3%) had been enrolled for school. Most of the participants (190, 76.8%) lived within 5 kilometres of the health facility (table 1). Thirty-four, 13.3% of the caretakers of the children were unemployed (table 2).

**Table 1:** Demographic characteristics of 255 children presumptive for pulmonary Tuberculosis attending health facilities in Kampala, 2015

Characteristic	Number (Percentage)
Health center attended	
Kisugu Health Center III	96 (37.7)
Kitebi Health Center III	54 (21.2)
Kawaala Health Center IV	80 (31.4)
Others *	25 (9.9)
Mean age in years ± SD	10.5 ± 3.5
Sex	
Male	110 (43.1)
Fem ale	145 (56.9)
Nationality of the child	
Ugandan	233 (91.4)
Congolese	6 (3.4))
Rwandese	9 (3.5)
Others**	7 (2.7)
Education level	
Not enrolled for school	17 (6.7)
Pre-Primary level	26 (10.2)
Primary level	164 (64.3)
Secondary level	48 (18.8)
Distance from health facility ***	
Less than 5 Kilometres	190 (76.8)
5 or more kilometres	58 (23.2)

Except where stated otherwise, values are numbers (percentage) of subjects

* Other health facilities include Kisenyi (18), Komamboga (6), and Kiswa (1)

** other nationalities included: Kenya and Somalia

*** 7 participants had missing information

**Table 2:** Characteristics of caretakers of 255 pulmonary Tuberculosis presumptive children attending health facilities in Kampala, 2015

Characteristics	Number (percentage)
Occupation of the caretaker	
Casual job as the source of income	180 (70.6)
Caretaker has a professional job	41 (16.1)
Caretaker is unemployed	34 (13.30
Median monthly income in Ugx, (IQR)*	150000 (90000, 250000)
Marital status	
Single	37 (14.5)
Married or cohabiting	186 (72.9)
Divorced or separated	21 (8.2)
Widow or widower	11 (4.3)
Education level	
Never went to school	19 (7.5)
Primary level	98 (38.4)
Secondary level	98 (38.4)
Tertiary level	40 (15.7)
Smoking status of the caretaker	
Not an active smoker or never smoked	243 (95.3)
Smokes for only a few days in a week	7 (2.7)
Smokes daily	5 (2.0)

Except where stated otherwise, values are numbers (percentage) of subjects.

* IQR inter-quartile range. The dollar rate at the time was 1USD = 2800 Ugx

### Household characteristics

Majority (235, 92.2%) of households used charcoal as source of energy and used outdoors in an open space as cooking area. Overall, 35 households (13.7 %) had a tobacco smoker. Twenty-eight children (11.0%) had had contact with an adult with confirmed TB (table 3).

**Table 3:** household characteristics of 255 pulmonary Tuberculosis presumptive children attending health facilities in Kampala, 2015

Characteristic	Number (percentage)
Mean persons per household ± SD	
Above 18 years	2.5 ± 1.4
Below 18 years	2.8 ± 1.6
Household has a smoker	35 (13.7)
Number of rooms in the house	
One room	72 (28.2)
Two rooms	90 (35.3)
Three or more rooms	58 (22.7)
Mean person density (persons per room) ± SD	2.6 ± 1.4
House has ventilators	236 (92.6)
Number of windows on the house	
No windows on the house	17 (6.7)
One window on the house	80 (31.4)
Two or more windows on the house	158 (61.9)
Mean windows per room ± SD)	0.9 ± 0.5
Cooking area	
Same room used for sleeping	24 (9.4)
Separate room used as kitchen	73 (28.6)
Separate building	12 (4.7)
Outdoors in an open place	146 (57.3)
Energy source for cooking	
Charcoal	235 (92.2)
Firewood	14 (5.5)
Others*	6 (2.2)
Child history of contact with TB patient	
Child had no contact with a TB patient	227(89.0)
Child had less frequent contact with a TB patient	21 (8.2)
Daily contact with a TB patient	7 (2.7)
Mean months of exposure to TB patient ± SD	4.8 (4.7)
Intensity of exposure	
Child Slept in the same bed with TB patient	9 (32.1)
Slept in same room, separate bed	4 (14.3)
Slept in same house, separate room	8 (28.6)
Slept in a separate house, but in the same neighbourhood	7 (25.0)

Except where stated otherwise, values are numbers (percentage) of subjects.

* Other sources of fuel include paraffin and electricity

Most children had been vaccinated for TB and had a BCG scar present. The most common symptom children presented with was chronic cough. Only 12 (4.7 %) were HIV positive while 60 (27.2 %) were stunted (table 4). Nine children were unable to provide a sputum sample.

**Table 4:** Clinical characteristics of 255 pulmonary Tuberculosis presumptive children attending public health facilities, Kampala, 2015

Characteristic	Number (percen tage)
Child was immunized against TB	240 (94.1)
Child has cough for more than two weeks	186 (72.9)
Symptoms suggestive of TB	
Frequent fevers	176 (69.0)
Drenching night sweats	147 (57.6)
Loss of appetite	149 (58.4)
Other symptoms (loss of appetite, weight loss, bloody sputum)	140 (54.9)
Child previously had TB (all completed treatment)	6 (2.4)
HIV status	
Negative	236 (92.6)
Positive	12 (4.7)
Do not know	7 (2.8)
Nutritional status based on Body mass index	
Underweight	140 (55.12)
Normal	108 (42.5)
Overweight	6 (2.4)
Nutritional status based on Weight-for-Age	
Wasted	5 (6.2)
Normal	85 (88.5)
Above normal	6 (6.3)
Nutritional status based on Height-for-Age	
Stunted	60 (23.6)
normal	185 (72.8)
Above normal	9 (3.5)
Weight-for-Height	
Malnourished	3 (13.6)
normal	14 (63.6)
Above normal	5 (22.7)

Except where stated otherwise, values are numbers (percentage) of subjects.

Burden of tuberculosis

The proportion of children with microbiologically confirmed PTB was 6.3% (95% CI: 0.9% – 11.6%), while for clinically diagnosed PTB, the prevalence was 7.5% (95% CI: 2.8% – 14.6%). For HIV positive and contacts, proportion was 41.7% (95% CI: 12.0% – 71.3%) and 89.3% (95% CI: 77.5% – 99.9%) respectively (table 5).

**Table 5:** prevalence of pulmonary tuberculosis among 255 children with clinical features suggestive of PTB attending KCCA health facilities, 2015

Categories	All-form TB		Smear positive PTB		Smear negative PTB	

	N	Proportion (CI)	N	Proportion (CI)	N	Proportion (CI)
Overall	35	13.7 (2.6 – 24.8)	16	6.3 (0.9 – 11.6)	19	7.5 (2.8 – 14.6)
HIV status						
Negative (236)	30	12.7 (0.6 – 24.8)	12	5.1 (0.9 – 10.3)	18	7.6 (0.4 – 15.7)
Positive (12)	5	41.7 (12.0 – 71.3)	4	33.3 (4.0 – 62.6)	1	8.3 (0.1 – 31.0)
Nutritional status						
Well nourished (194)	22	11.3 (4.5 – 18.2)	8	4.1 (0.1 – 13.2)	14	7.2 (1.2 – 13.2)
Malnourished (60)	13	21.7 (0.1 – 53.1)	8	13.3 (0.5 – 22.0)	5	5.3 (0.5 – 13.0)
Contact with TB patient						
No contact (227)	29	10.7 (1.0 – 22.5)	10	21.4 (1.4 – 43.0)	19	8
Had contact (28)	25	89.3 (77.5 – 99.9)	25	78.6 (57.0 – 99.9)	0	No Observation

### Factors associated with Pulmonary Tuberculosis

When children with both microbiologically confirmed and clinically diagnosed PTB were considered together in multivariate logistic analysis, number of adults in the household (aOR = 1.4; 95% CI: 1.10 – 1.76), having a smoker in the household (aOR = 1.6; 95% CI: 1.07 – 6.86), and being malnourished (aOR = 2.2; 95% CI: 1.01 – 4.15) were associated with PTB (table 6).

**Table 6:** Factors predicting all-form tuberculosis among 255 children attending public health facilities in Kampala, 2015

Characteristics	Crude OR (95% CI)	P value	Adjusted OR (95% CI)	P value
Number of adults at home	1.3 (1.08 – 1.64)	0.008	**1.4 (1.10 – 1.76)**	**0.006**
Presence of a smoker				
No smoker at home	Reference		Reference	
There is a smoker at home	0.3 (0.14 – 0.74)	0.008	**1.6 (1.07 – 6.86)**	**0.035**
HIV status				
HIV negative	Reference		Reference	
HIV Positive	4.9 (1.46 – 16.45)	0.010	2.7 (0.54 – 13.11)	0.230
Nutritional Status				
Well nourished	Reference		Reference	
Child is stunted	2.2 (1.01 – 4.62)	0.046	**2.2 (1.01 – 4.15)**	**0.049**

* INH Isoniazid Preventive therapy

When children with microbiologically confirmed PTB were analyzed separately, number of windows per room (aOR = 0.1; 95% CI: 0.01 – 0.48), history of contact with an adult having confirmed TB (aOR = 8.3; 95% CI: 2.02 – 33.70) and stunting (aOR = 3.6; 95% CI: 1.28 – 9.99) were associated with PTB (table 7).

**Table 7:** Factors predicting smear-positive pulmonary tuberculosis among 255 children attending public health facilities in Kampala, 2015

Characteristics	Crude OR (95% CI)	P value	Adjusted OR (95% CI)	P value
Number of adults at home	0.6 (0.34 – 1.06)	0.080	0.6 (0.32 – 1.18)	0.140
Persons per room	1.1 (0.74 – 1.52)	0.742	**0.1 (0.01 – 0.48)**	**0.001**
Contact with TB patient				
No contact	Reference		Reference	
Had contact with TB patient	5.9 (1.96 – 17.83)	0.002	**8.3 (2.02 – 33.70)**	**0.003**
HIV status				
HIV negative	Reference		Reference	
HIV Positive	9.3 (2.46 – 35.41)	0.001	2.5 (0.75 – 8.54)	0.133
Nutritional Status				
Well nourished	Reference		Reference	
Child is stunted	3.9 (1.35 – 11.30)	0.012	**3.6 (1.28 – 9.99)**	**0.015**

*INH Isoniazid Preventive therapy

Children with clinically diagnosed PTB were separately analyzed. Staying more than 5 kilometers from the health facility offering TB services (aOR = 3.1; 95% CI: 1.02 – 9.66), having a tobacco smoker in the household (aOR = 8.1; 95% CI: 2.72 – 24.14) and using firewood as energy source for cooking (aOR = 15.7; 95% CI: 2.30 – 106.56) were associated with PTB (table 8).

**Table 8:** Factors predicting clinically diagnosed tuberculosis among 255 children attending public health facilities in Kampala, 2015

Characteristics	Crude OR (95% CI)	P value	Adjusted OR (95% CI)	P value
Distance from Clinic				
Less than 5 km	Reference		Reference	
5 or more Km	2.3 (0.83 – 6.12)	0.109	**3.1 (1.02 – 9.66)**	**0.045**
Presence of a smoker				
No smoker at home	Reference		Reference	
There is a smoker at home	0.2 (0.07 – 0.48)	0.001	**8.1 (2.72 – 24.14)**	**<0.001**
Energy source				
Charcoal	Reference		Reference	
Firewood	1.1 (0.13 – 8.57)	0.962	**15.7 (2.30 – 106.6)**	**0.005**
Stove and electricity	6.8 (1.16 – 40.24)	0.033	0.8 (0.09 – 7.39)	0.857

Only 5.3% (1/19) of the smear negative TB children and 81.3% (13/16) of the smear positive children were initiated on treatment within a month of diagnosis.

## Discussion

In this study we determined the burden and predictors of TB, and how a standardized approach to clinical diagnosis (using the Union Pediatric TB Desk Guide) could impact case notification and treatment among older children in Uganda. We found that 13.7 % of the children we enrolled into our study (with presumed TB) had clinical or smear positive TB. The burden of TB was higher among the malnourished, HIV positive and children with history of contact with an adult TB patient.

Previous studies have reported lower prevalence of TB among children 23 2, 9 24. However, the lower prevalence was not among children with presumed TB. Kampala, the location for our study, is over-crowded yet crowding has been shown to increase risk of TB infection 25, 26. The lower prevalence in national notification figures could be due to poor recording and reporting of routine pediatric TB cases in the national TB registries which has a lot of missing information and mainly bases on smear results 10, 27. These findings may imply that the burden of childhood TB has been undermined.

Our findings showed that more than half of the children with TB were missed by the routine diagnostic practices, more so children with smear-negative TB. The high rate of missed TB diagnosis can be attributed to the difficulty in diagnosis of TB, yet in peripheral facilities patient management including diagnosis and treatment is routinely done by lower medical cadres including clinical officers and nurses. Also, the clinical algorithms for guiding the diagnosis of TB are not routinely used during the management of these children. Lack of capacity to diagnose TB had been previously highlighted as a barrier to diagnosis of TB in Uganda 27. Furthermore, there is a significant loss to follow-up of patients with TB. In our study, only 40% of children diagnosed with TB being started on treatment within a month following diagnosis.

Household overcrowding, indoor air pollution and malnutrition were significantly associated with TB spread among children in our study. Malnutrition impairs the body's immunity making the child more susceptible to TB 25 on the other hand, a child who develops tuberculosis is more likely to develop malnutrition 5. With high rates of malnutrition among the children in Uganda, TB is likely to continue being a major cause of morbidity. Indoor air pollution has been associated with development of PTB since pollutants impair mucosal integrity and lower surface immunity 5, 25. This is in line with the findings from our study since having a tobacco smoker in the household was associated with a 1.6 increase in the odds of developing PTB 5.

Our study had several strengths. Frist, while many studies among children report one form of TB, in our study we report both laboratories confirmed and clinically diagnosed TB. In addition, we evaluated TB diagnosis and management using a standard guideline. Moreover, we did our evaluation retrospectively when the actual patient diagnosis was already done. This way, our presence did not influence how clinicians diagnose the patients. The retrospective evaluation enabled us to evaluate how well TB is diagnosed but also gave us time to determine the proportion of children diagnosed with TB that were started on treatment. Our study enrolled participants from six public health facilities, which makes our sample representative of the general population. Also, we employed robust analysis methods and controlled for several factors that could confound the association between TB and our main predictors. However, our study had some limitations. Children below five years were not included in the study. This is a special population with regards to TB. Additionally, the study used hospital participants who were already presumed to have TB; therefore, they were more likely to have TB than the general population. This population is more likely to over-estimate the burden of TB. These shortcomings could have introduced a selection bias. For some of the characteristics like monthly income, and BCG vaccination, the researcher had to rely on self-report. This may have introduced an information bias in the study. In the study sputum cultures, the gold standard for TB diagnosis, were not done. Therefore, it is possible that some of the TB cases were missed.

## Conclusions

Clinical TB among older children is underdiagnosed and undertreated. There is pending availability of more sensitive and specific diagnostic tests, need ways to disseminate and promote uptake of standardized clinical algorithms. There is a need to strengthen contact TB tracing so that such cases can be actively detected even at community level. Larger studies, employing gold standard diagnostic means is recommended to further study the pediatric TB burden, especially among contacts to TB patients, children below 5 years, and the malnourished children.

## Abbreviations

BCG: Bacille Calmette-Guérin
BMI: Body Mass Index
EIA: Enzyme Immuno Assay
FM: Fluorescent Microscopy
HIV: Human Immuno-deficiency Virus
IPT: Isoniazid Preventive Therapy
KCCA: Kampala Capital City Authority
MoH: Ministry of Health
NTLP: National Tuberculosis and Leprosy Program
NTRL: National Tuberculosis Reference Laboratory
OR: Odds Ratio
PHC: Primary Health Care
PTB: Pulmonary Tuberculosis
TBL: Tuberculosis
ZN: Zeihl Neelsen
